# The roles of the microtubule cytoskeletal network in cardiac mechanobiology

**DOI:** 10.1242/jcs.264308

**Published:** 2026-03-23

**Authors:** Jack F. Murphy, Xuan Li

**Affiliations:** ^1^ Department of Engineering, University of Cambridge, Cambridge CB2 1PZ, UK; ^2^ Victor Phillip Dahdaleh Heart and Lung Research Institute, University of Cambridge, Cambridge Biomedical Campus, Cambridge CB2 0BB, UK

**Keywords:** Cytoskeleton, Mechanobiology, Cardiomyocytes

## Abstract

The intracellular cytoskeletal network is a central mediator of mechanobiology, transmitting extracellular cues from the cell membrane to elicit downstream changes in cell behaviour and morphology. In cardiomyocytes, mechanotransduction via the intracellular cytoskeleton plays a pivotal role in regulating cardiac output, development and pathological remodelling. Various structural components contribute to this process, including tension-mediated ion channels, contractile sarcomere units, transmembrane cell junction proteins, intermediate filaments and the microtubule network. Among these components, microtubules are the largest and most rigid cytoskeletal elements. This Review highlights the essential, but often understated, role of microtubules in cardiomyocyte mechanobiology, particularly in their coordination with other cytoskeletal elements. Furthermore, this Review highlights how the microtubule cytoskeletal network is connected to cardiac disease phenotypes.

## Teaser

**Summary:** Microtubules are essential cytoskeletal regulators of cardiomyocyte mechanobiology, coordinating with other cytoskeletal elements to govern both physical properties and mechanotransduction responses.

## Introduction

Mechanobiology offers insights into how cells sense and respond to mechanical stimuli such as contraction, compression, stretching, tension and shear stress, thereby influencing cell development, physiology and disease phenotypes. These mechanical stimuli are propagated downstream, modifying cell behaviour and resulting in structural remodelling, functional changes and cell mobility. Mechanobiology is particularly important in cardiac tissues, where cells operate in a constant contractile environment and must respond rapidly to varying load conditions. Cardiomyocytes continually adjust their contraction velocity, beating frequency and contractile force in response to changing biophysical needs. For example, the average human heart rate increases from a resting baseline of 60–100 beats per minute to ∼130–150 beats per minute when exercising. This change is driven by a physiological need for increased oxygen in tissues around the body and is mechanically controlled through adjustments in cardiomyocyte contractility. The myocardium, the muscular layer of the heart wall, whose volume is primarily composed of cardiomyocytes, is a highly specialised environment where mechanical efficiency is paramount. In cardiomyocytes, the mechanobiology is uniquely bi-directional; as in other cell types, the environment impacts their biology, but cardiomyocytes also self-contract, thereby introducing active forces back onto their surrounding matrix and neighbouring cells. If cardiomyocytes are subjected to volumetric compression through uniform forces on all sides, the cells might experience a volume change that can alter cell behaviour, cytoskeletal organization, ion channel regulation and cardiomyogenesis (Deaton, 1997; Happe and Engler, 2016; Faure, Venturini and Roca-Cusachs, 2025). Pressure and volume overload conditions can lead to disease pathologies and increased arrhythmia risk (McCain and Parker, 2011; Happe and Engler, 2016; Yu et al., 2022). Therefore, the forces received by cardiomyocytes drastically modify their outcome, and aberrant signalling in mechanotransduction pathways can have detrimental effects, contributing to cardiomyopathies (Jaalouk and Lammerding, 2009). One contributing factor to the pathologies of heart diseases is a disruption of the force transmission network between the extracellular matrix (ECM), cytoskeleton and nucleus (Jaalouk and Lammerding, 2009). It has been well-established for over 30 years that the cytoskeleton plays a vital role in modulating mechanotransduction pathways (Wang et al., 1993).

**Fig. 1.**
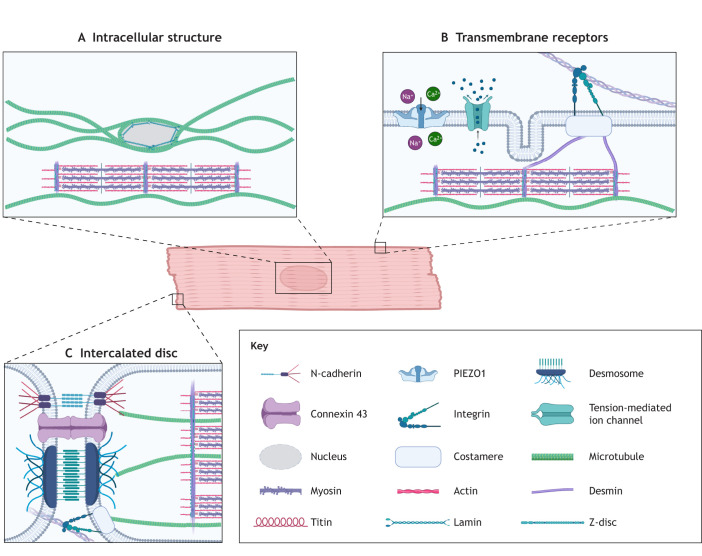
**Cytoskeletal structures associated with cardiomyocyte mechanobiology.** (A) Intracellular cardiomyocyte structures involved in mechanotransduction pathways. Microtubules are anchored to desmins at the Z-discs of sarcomeres and form a cage surrounding the nucleus. Lamins form a dense network on the inner surface of the nuclear envelope. (B) Transmembrane mechanotransduction receptors. PIEZO1 and other tension-mediated ion channels control the influx and efflux of particles that regulate cardiomyocyte contraction. Adhesion junctions, such as integrins at costameres, provide mechanosensation of the surrounding ECM. The intermediate filament desmin links to the costamere and forms a network connecting Z-discs of adjacent myofibrils. (C) Intercalated disc junctions that attach cardiomyocytes to provide electro-mechanical coupling and allow cardiac tissue to function as one continuum. These junctions are composed of adherens junctions, desmosomes and gap junctions. Created in BioRender by Murphy, J., 2026. https://BioRender.com/5uiseci. This figure was sublicensed under CC-BY 4.0 terms.

**Fig. 2.**
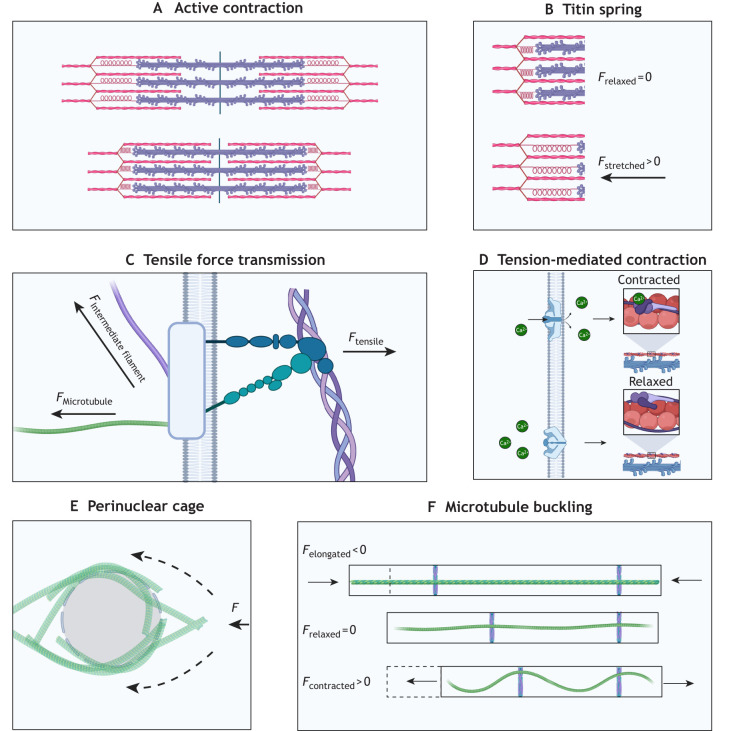
**Structural components for mechanosensing in a mature cardiomyocyte.** (A) Sarcomere shortening. Actin–myosin motor interactions shorten sarcomere units, thus causing hierarchical shortening at the cell and tissue level. (B) Titin elasticity. The elastic titin spring provides passive tension to resist overstretching of the sarcomere. (C) Transmembrane force transmission. Integrins and other adhesion complexes relay tensile signals intracellularly from the extracellular matrix. (D) Mechanosensitive ion channels. Tension-mediated ion channels regulate cardiomyocyte contraction through controlling Ca^2+^ tropomyosin binding and regulation of action potential. (E) Perinuclear microtubule cage. A dense microtubule network surrounds the nucleus, shielding it from mechanical loads and redistributing forces throughout the cell. (F) Microtubule buckling. Microtubules anchored at Z-discs undergo buckling during contraction. This buckling opposes sarcomere contraction and elongation, providing structural rigidity and a restoring force toward the relaxed length. Created in BioRender by Murphy, J., 2026. https://BioRender.com/5uiseci. This figure was sublicensed under CC-BY 4.0 terms.

Mature cardiomyocytes are elongated and rod-shaped, with one or two centrally located nuclei in mammals. The highly organized cardiomyocyte structure enables the heart to contract rhythmically and continuously throughout life. A defining structural feature of cardiomyocytes is the presence of sarcomeres, the repeating contractile units. The cardiomyocyte cytoskeleton is present in the sarcomere and greatly impacts how mechanical forces are received (Fig. 1). The cytoskeleton comprises active components that generate contraction and passive components that regulate it. The active machinery, explained through sliding filament theory (Powers et al., 2021), consists of two parallel filaments – thick filaments (mainly composed of the protein myosin) and thin filaments (mainly composed of the protein actin), which partially overlap when relaxed. During contraction, motility is generated by the collective action of many myosin heads being briefly bound to actin, generating power strokes that shorten the cell (Fig. 2A). Titin, a giant sarcomere protein, functions as a spring connecting thick filaments to the Z-discs and provides passive tension to resist overstretching of the sarcomere (Helmes et al., 1996; Granzier and Labeit, 2004) (Fig. 2B). The Z-discs anchor thin filaments and are tethered to myosin via titin to provide myocardial elasticity, whereas the M-band centrally anchors the thick filaments. The cardiac Z-disc has increasingly been recognised as a key junction for signal transduction rather than serving strictly as a structural component (Frank and Frey, 2011). Sarcomere contraction is tightly regulated by Ca^2+^. T-tubules are inward extensions into the cell membrane that transmit electrical signals into the cell and coordinate Ca^2+^ exchange. Intermediate filament proteins maintain structural integrity and organisation. Desmin and lamins are the most abundant intermediate filament proteins in adult cardiomyocytes; together, they facilitate the efficient and safe transmission of mechanical cues to the nucleus, Z-discs and cell junctions (West et al., 2023). Intermediate filaments also regulate organelle positioning by tethering organelles in place (West et al., 2023). An often-overlooked component of the cardiac cytoskeletal network is the microtubule. They are the longest, straightest and most rigid cytoskeletal components, and largely determine myocardial stiffness by providing strong mechanical resistance and producing passive force during cardiomyocyte contraction. These passive forces generated by microtubules are essential to balance the contraction–relaxation cycle and provide mechanical stability (Robison et al., 2016; Caporizzo et al., 2018; Yu et al., 2021). Importantly, microtubules are key mediators of intracellular protein transport, signal transduction and mechanosensation (Seetharaman et al., 2022), and changes in the microtubule network are observed in heart disease pathologies (Yu et al., 2021; Caporizzo and Prosser, 2022; Warner et al., 2022).

Throughout this Review, we aim to provide a current understanding of cardiomyocyte mechanobiology, emphasizing the role of microtubules, in regulating mechanotransduction pathways and cell fate. We also briefly discuss how the mechanobiology of other cardiovascular cells impacts heart disease.

## Mechanobiology in cardiomyocytes

In cardiomyocytes, mechanobiology guides structural development and regulates contractile function throughout the lifetime, linking cellular mechanics directly to heart function. Starting during embryonic development, when cardiomyocytes differentiate and mature, this regulation continues lifelong as cells adapt to physiological and pathological changes in the heart.

### Cardiomyocyte differentiation

During development, cardiomyocytes mature into highly specialized actuators capable of strong, synchronous contractions. Immature cardiomyocytes differ markedly from mature cardiomyocytes, as shown by differences in morphology, metabolic profiles, cytoskeletal organization and electrophysiology (Karbassi et al., 2020; Cho et al., 2022). During development, mammalian cardiomyocytes shift from an immature, proliferative, diploid and mononucleated state to a mature, polyploid and often binucleated phenotype (Elia et al., 2023). During mitosis, microtubules form the mitotic spindle, which segregates chromosomes and enables cell division. As cardiomyocytes become mature, they exit the cell cycle. In mature cardiomyocytes, the mitotic spindle no longer anchors the actomyosin ring to the plasma cell membrane (Leone et al., 2018). Although the physiological relevance of binucleation remains unclear, the proportion of binucleated cells can be as high as 90% in some mammalian species (Elia et al., 2023), indicating that binucleation is an important feature of the mature cardiomyocyte phenotype.

The difference between mature and immature phenotypes has been highlighted by the emergence of induced pluripotent stem cell-derived cardiomyocytes (iPSC-CMs). First developed in 2006, iPSCs provide near-limitless access to pluripotent stem cells that can be guided into cardiomyocyte-like cells (Takahashi and Yamanaka, 2006; Turnbull et al., 2018). Although iPSC-CMs have broad potential in drug screening pipelines and allow for investigation into novel therapeutics, they continue to suffer from many limitations – for example, they display a distinctly immature phenotype (Cho et al., 2022; Zhuang et al., 2022), which reduces the translational efficacy of results obtained using them as a model (Cho et al., 2022). Mechanobiology is closely tied to the cardiac maturation process because contractile mechanical stresses generated by electrical conditioning ‘train’ developing cells throughout maturation, which guides them to assemble the robust aligned sarcomeres and Ca^2+^-handling systems essential for powerful adult contraction (Radisic et al., 2004). Cyclic stretching improves iPSC-CM maturation through mechanotransduction (Song et al., 2022). iPSC-CMs cultured on a cyclical substrate stretch display a more mature phenotype than those in static culture by having improved cell alignment, elongated sarcomeres and cell shape and enhanced mRNA expression of maturation markers (Song et al., 2022). Similar stretching-induced elongation is observed in neonatal rat cardiomyocytes, along with increased expression and polarised localisation of the gap junction protein connexin-43 (Cx43; also known as GJA1) at cell ends (Salameh et al., 2010). Cyclic stretch also upregulates nuclear mechanotransduction genes such as those encoding Yap1, lamins, plectin and desmin (Song et al., 2022), and both sarcomere structures and cell shape align via cellular tension (Russell et al., 2010), indicating that these morphological and physiological improvements are closely tied to established mechanotransduction pathways. Electrical pulsing to stimulate cardiomyocyte contraction can similarly enhance cardiomyocyte maturation. Chronic electrical conditioning of immature cardiomyocytes results in elongated cells, enhanced intercalated disc and gap junction expression and improved sarcomere alignment (Radisic et al., 2004). Notably, the profile of electrical stimulation significantly modifies the resulting cardiomyocyte phenotype – a low ramp-up in frequency can restore a positive force–frequency relationship, a hallmark of mature cardiomyocytes that is often lacking in immature models (Zhao et al., 2020). The positive force frequency relationship seen in mature cardiomyocytes means that as they beat faster, they also beat stronger to compensate for the increased load required. The development of mechanobiology during the maturation process will, conversely, promote the electrical maturation of a beating cardiomyocyte.

Notably, the mechanical environment greatly changes through different stages of development (Engler et al., 2008; Jacot et al., 2010). The adult myocardium is roughly ten times stiffer than the embryonic myocardium (Majkut et al., 2013; Ward and Iskratsch, 2020). This increase in stiffness is largely due to heightened collagen crosslinking and enhanced cardiomyocyte maturation, such as improved sarcomere organisation (Badenhorst, 2003). Cardiomyocytes perform optimally on substrates with stiffness that matches their physiological environment – embryonic or immature cells prefer and are adapted for softer substrates than are adult cells. A mismatch between cellular state and environmental stiffness can impair contraction dynamics (Engler et al., 2008). Intracellular physical modifications have been explained by myosin-driven contraction sensing and adapting to the elasticity of the surrounding tissue (Barrick and Greenberg, 2021), whereas tissue-level physical properties are largely dependent on the composition and organisation of the ECM. Altered ECM stiffness changes individual cardiomyocyte tension and modulates tissue-level conduction through gap junction redistribution. The ECM is composed of structural proteins, such as laminin, fibronectin and collagen, which collectively determine matrix elasticity through their density, orientation and degree of crosslinking. Collagen is a primary contributor to matrix stiffness in the heart, with higher collagen concentration leading to increased stiffness (Zile et al., 2015). Other ECM proteins, such as fibronectin and laminin, serve as focal adhesion points for integrins on the cardiomyocyte surface (Samarel, 2005). Differentiation in response to alterations in the mechanical environment has been linked to changes in transcriptional regulators YAP1 and TAZ (collectively, YAP/TAZ; TAZ is also known as WWTR1). For example, a stiff ECM promotes YAP/TAZ nuclear localization, where they activate gene expression programmes that drive maturation and sarcomere assembly (Ragazzini et al., 2022).

### Cardiomyocyte function

In the adult heart, mechanobiology is important for normal cardiac function by maintaining proper contraction–relaxation cycles, electrical stability and adapting to workload changes. A cardiomyocyte's primary function is to act as a mechanical actuator that contracts to pump blood throughout the body. This central role is reflected in how we assess cardiac function: for example, ejection fraction, the percentage of blood that is pumped out with each heartbeat, is a purely mechanical quantification of cardiac efficiency and performance.

Force transmission occurs at multiple levels: from larger-scale cell–cell and cell–ECM junctions to intracellular mechanosensors, including the cytoskeletal network and the nucleus, which are interlinked with the contractile unit, the sarcomere. Cardiomyocytes are attached to the surrounding ECM by focal adhesions, such as integrins, and to each other by specialized cell–cell junctions that contain gap junctions, desmosomes, adherens junctions and costameres, which are structures that connect the internal contractile cardiomyocyte machinery to the surrounding cells (Samarel, 2005). Intracellularly, they connect to the cytoskeletal network, including actin filaments, microtubules and intermediate filaments (Fig. 2C). The forces transmitted through these junctions can reach sarcomere-based mechanosensors, such as titin (Puchner et al., 2008; Bogomolovas et al., 2014), influencing contraction. The elastic titin protein signalling is force dependent, with mechanical force exposing sites hidden (cryptic) at low forces but exposed by force-induced unfolding (Alegre-Cebollada et al., 2014; Bianco et al., 2015). Extracellular mechanical forces are also transduced to nuclear responses via the linkers of nucleoskeleton and cytoskeleton complex (LINC) (Heffler et al., 2020). Cardiomyocyte nuclei are mechanically vulnerable due to large forces and constant contractile strain. For this reason, proper force transmission through the LINC complex is required to maintain nuclear structure and genome organisation; when the LINC complex is disrupted, cardiomyocyte function is severely altered with possible nuclear collapse (Heffler et al., 2020). Atypical force transmission to the nucleus can also result in changes to gene expression and nuclear morphology (Coscarella et al., 2023). Myosin contraction and mechanotransduction are closely tethered (Barrick and Greenberg, 2021). Tension generation by myosin molecules is required to maintain sarcomere orientation. The disruption of myosin-mediated contraction via blebbistatin, a small molecule that selectively inhibits myosin motor for muscle contraction, leads to a loss of sarcomeres in iPSC-CMs (Chopra et al., 2018; Barrick and Greenberg, 2021). Blebbistatin also reduces mechanosensation in neonatal rat cardiomyocytes and has been shown to extend culture viability of adult cardiomyocytes (Kabaeva et al., 2008; Pandey et al., 2018). Actomyosin contractile machinery is optimized for the stiffness of the tissue in which the cardiomyocyte is embedded in; either an increase or a decrease in tissue stiffness impairs contractility (Chen et al., 2018; Barrick and Greenberg, 2021). This demonstrates that healthy cardiomyocytes self-specialize and adapt their cytoskeletal profile to match the mechanical properties of their environment.

Another important example of innate mechanobiology regulation is the PIEZO channel family, a group of mechanosensitive ion channels present in mammalian cells (Fang et al., 2021), which, in cardiomyocytes, control crucial processes, such as stretch-activated Ca^2+^ influx (Liang et al., 2017; Zhang et al., 2021). PIEZO channels open and close in response to mechanical stress and, in cardiomyocytes, are functionally tethered to the actin cytoskeleton (Wang et al., 2022). These channels facilitate the transmembrane movement of Na^+^, K^+^ and Ca^2+^ ions, all of which directly impact cardiomyocyte contractile machinery and are essential for healthy cardiac function (Coste and Delmas, 2024) (Fig. 2D). Together, these interconnected mechanosensory systems allow cardiomyocytes to maintain mechanical homeostasis and quickly adjust to physiological demands.

## Microtubule roles in cardiomyocyte mechanobiology

For over 30 years, it has been established that the cytoskeletal network is a primary component of cellular mechanotransduction (Wang et al., 1993). In cardiomyocytes, this network consists of spectrin, microtubules, actins and intermediate filaments, and is well organized in the base contractile units known as sarcomeres. These components are essential for maintaining the ability of cardiomyocytes to function as biomechanical actuators, and their dysregulation can lead to heart failure (Sequeira et al., 2014). Actin and its motor protein, myosin, constitute the active machinery needed to power cardiomyocyte contraction. Structural proteins that make up the Z-discs and M-lines, such as myomesin, titin and α-actinin family proteins, support this active machinery and ensure the proper parallel orientation needed for effective actomyosin motor interactions and coordinate cell- and tissue-level contractions (for a more detailed discussion of the sliding filament theory, see Powers et al., 2021). Spectrin stabilises the cell membrane and anchors membrane ion channels during the contraction–relaxation cycles (Unudurthi et al., 2018). Intermediate filaments, such as lamin and desmin, tether organelles in place and provide structural support to the cell (Tsikitis et al., 2018; West et al., 2023). Unlike actin filaments, which provide moving tracks for contraction, or intermediate filaments, which provide structural continuity, microtubules primarily modulate passive resistance, viscoelasticity and intercellular trafficking. The microtubules influence the physical layout, mechanical properties and contraction dynamics, and they tightly regulate macromolecular shuttling and modulate signalling cascades (Caporizzo and Prosser, 2022; Warner et al., 2022). In cardiomyocytes, microtubules serve functions far beyond being dynamic structures for cell division, cell motility and intracellular transport; they are multifunctional structures that are key for maintaining healthy cardiac function. Below, we describe microtubule-dependent mechanisms that influence cardiomyocyte structure, contractility and signalling.

### Microtubules in cardiomyocyte structure

Microtubules are not only structural tracks for intracellular transport; in cardiomyocytes they form a mechanical scaffold that integrates maturation, structural and mechanotransductive processes. Microtubules form a dense, interconnected network within cardiomyocytes, helping to distribute mechanical stresses throughout the cell. Microtubules are necessary for the formation of mature binucleated, polyploid cardiomyocytes (Leone et al., 2018). The microtubule network pattern in immature cells, such as iPSC-CMs, differs substantially from that of mature cardiomyocytes. These differences likely reflect both the immature phenotype of iPSC-CMs and the fact that microtubules are one of the later cytoskeletal structures to mature, continuing to develop postnatally (Cartwright and Goldstein, 1985), implicating microtubules progressively adapt to fulfil their specialized roles in mature cardiomyocyte function. Microtubules are essential for establishing and maintaining mature cardiomyocyte structure, and alterations in the microtubule network are observed in multiple mechanosensitive-related disease phenotypes, including hypertension (LeBar et al., 2024), dilated cardiomyopathy (Dees et al., 2012), ischemic failing hearts (Yu et al., 2021) and Duchenne muscular dystrophy (Khairallah et al., 2012).

Microtubules mechanically resist compression during contraction, directly influencing contraction amplitude. They attach to various cytoskeletal components, including cell junctions, t-tubules and sarcomere Z-discs, as well as forming a perinuclear cage in the mature cells, which can help to mechanically protect the nucleus and organise intracellular transport (Caporizzo and Prosser, 2022) (Fig. 2E). The biochemical and mechanical properties of microtubules can be modified through post-translational modifications that alter their physical structure and binding interactions (Caporizzo and Prosser, 2022; Warner et al., 2022). Microtubule detyrosination, the enzymatic removal of tyrosine from the α-tubulin tail, generates more-stable long-lived microtubules and increases cellular stiffness (Robison et al., 2016). In cardiomyocytes, the detyrosinated microtubules are densely enriched within the perinuclear microtubule network (Yu et al., 2021), which is resistant to depolymerisation and might function as effective force transducers. This perinuclear microtubule network maintains nuclear position and protects the nucleus from deformation during the contractile cycle; disruption of the microtubule network leads to nuclear elongation, which can impact gene expression and DNA damage (Heffler et al., 2020).

The microtubule network must remain well-organised within cardiomyocytes to ensure proper functionality. During cardiomyocyte maturation, the centrosome, which serves initially as a microtubule-organising centre (MTOC), undergoes dramatic structural reorganisation. During this process, centrosome components relocate from the centriole to the nuclear envelope in a process known as centrosome reduction, a developmentally programmed event associated with cell cycle exit. In infantile dilated cardiomyopathy, however, the centrosome components remain abnormally localised at the centriole, leading to subsequent microtubule network defects (Chun et al., 2023). The developmental pathways and cues that coordinate microtubule reorientation during cardiomyocyte maturation remain largely unexplored.

To contract as a mechanically continuous tissue, cardiomyocytes must be properly coupled to adjacent cells to ensure effective excitation–contraction propagation. Microtubules organise the sarcomere structure and position key organelles, such as mitochondria and sarcoplasmic reticulum, while providing a rigid anisotropic structure to the cardiomyocytes and localising proteins at key locations, including at t-tubules and intercalated discs (Uchida et al., 2022). This highly ordered microtubule architecture is essential for efficiently translating action potentials into physical contraction.

### Contraction dynamics

Cardiomyocytes undergo continuous contraction–relaxation cycles throughout their lifespan. The active contractile machinery relies on interactions between actin and myosin, regulated by cytoplasmic Ca^2+^ influx. Although actin–myosin interactions generate active contractile force, microtubules tune how much and how fast a cardiomyocyte can shorten and re-lengthen by imposing passive viscoelastic resistance (Caporizzo et al., 2018). Microtubules, the most rigid cytoskeletal filaments, directly influence the contraction dynamics of cardiomyocytes (Caporizzo et al., 2018; Pollard and Goldman, 2018). Microtubules are not part of the active contraction powered by actomyosin motor complexes. Instead, the microtubule cytoskeleton constitutes a passive contractile machinery that regulates contraction and relaxation during each myocyte contraction cycle. Microtubules provide a viscoelastic resistance to cardiomyocyte motion and imbue a solid mechanical rigidity (Caporizzo et al., 2018), contributing to both the viscous and elastic components in the biomechanical models of heart tissue (LeBar et al., 2024; Tajvidi Safa et al., 2024). Because microtubules resist sarcomere shortening through buckling, and sarcomere elongation through stretching, their stiffness directly modulates contraction amplitude and the kinetics at systole and diastole (Robison et al., 2016).

Because they are anchored at Z-discs of the sarcomere, microtubules are passively forced into repetitive bending and buckling modes during each contraction, where they provide physical resistance force that opposes the positive sliding force generated by actin-myosin interactions that shorten the sarcomere (Robison et al., 2016). This passive resistance generated by microtubules also determines the extent of cardiomyocyte relaxation (Fig. 2F). Repetitive bending and relaxing, together with microtubule damage promoted by injury signalling, could potentially lead to microtubule fatigue (Nasrin et al., 2024), although this process has not been studied in cardiomyocytes. Microtubule repair processes, which remain largely unidentified, might contribute to increased stiffness. These considerations highlight that microtubule integrity is not static but dynamically maintained, emphasising the importance of cytoskeletal maintenance.

Microtubules adopt three predominant orientations in human cardiomyocytes (Uchida et al., 2022): the first and most predominant subpopulation aligns along the longitudinal axis, the second subpopulation forms a perinuclear cage and the third subpopulation wraps around the short axis. Their alignment and organization significantly impact mechanical performance, with aligned crosslinked microtubules generating significantly more force per strain than coiled or uncrosslinked microtubules (Caporizzo and Prosser, 2022). Detyrosinated microtubules are more stable and stiffer than their tyrosinated counterparts (Salomon et al., 2022). Mechanically, microtubules primarily maintain cell shape homeostasis and resist deformation; they buckle during contraction and stretch during elongation, providing a restoring force that returns the cardiomyocyte to its relaxed size. Healthy cardiomyocytes contain both tyrosinated and detyrosinated microtubules. Detyrosinated microtubules are laterally anchored by desmin at Z-discs and therefore buckle in short steep deflections (Robison et al., 2016), whereas tyrosinated microtubules, lacking this lateral anchoring, have a gentle deflection arc over their whole length. The lateral anchoring and short buckling wavelength mean that detyrosinated microtubules provide a much larger restoring force during systole than tyrosinated microtubules (Robison et al., 2016; Uchida et al., 2022). In failing cardiomyocytes, increased microtubule detyrosination increases stiffness and impedes contraction (Caporizzo and Prosser, 2022; Salomon et al., 2022; Uchida et al., 2022; Yu et al., 2021). These modifications also impair diastolic stretch in the myocardium, which leads to heart failure (Caporizzo et al., 2020; Yu et al., 2021; Eaton et al., 2024). The regulatory enzymes for microtubule detyrosination have been identified for controlling cardiomyocyte contractility (Yu et al., 2021; Warner et al., 2022; Eaton et al., 2024). For example, the enzymes directly regulating the microtubule detyrosination–tyrosination cycle include tubulin tyrosine ligase (TTL) and tubulin carboxypeptidases (TCPs) (Warner et al., 2022). TTL adds a tyrosine residue to the α-tubulin tail of microtubules, whereas TCPs, such as vasohibins, remove the tyrosine from the C-terminal of α-tubulin tail.

In addition to elasticity, microtubules contribute to cardiomyocyte viscosity. Their adaptable and breakable crosslinking to other cytoskeletal components provides viscous resistance to length changes during both contraction and relaxation phases (LeBar et al., 2024). This viscosity is highly rate-dependent – microtubules provide minimal mechanical effects at low strain rates but dominate mechanical resistance at high strain rates, consistent with the dynamics of physiological contraction (Caporizzo et al., 2018).

### Intracellular transport and signalling modulation

The contributions of microtubules to cardiac performance go far beyond purely mechanical roles; they are also essential regulators of biochemical signalling pathways and protein transport. The densely packed sarcomere organisation in cardiomyocytes limits diffusion-based protein and molecular transport, and microtubules are the predominant cytoskeletal component responsible for proper localisation through active transport of proteins and mRNAs (Scholz et al., 2006; Macquart et al., 2018; Uchida et al., 2022).

One key mechanism for protein localisation is localised translation at ribosomes evenly spaced along sarcomere units (Haddad et al., 2024). Knockout of ribosomal protein SA (encoded by *RPSA*), a Z-disc localised protein in cardiomyocytes, results in a mislocalisation of ribosomes that leads to disrupted translation, and ultimately contributes to disease phenotypes such as dilated cardiomyopathy (Haddad et al., 2024). Crucially, this essential, strict organisation has been shown to be microtubule dependent (Haddad et al., 2024).

Microtubules also mediate protein localisation through direct intracellular transport. For example, Cx43, a key gap junction protein, polarises to the intercalated discs in cardiomyocytes and is crucial for cell–cell communication. Disruption of the microtubule network can lead to a form of dilated cardiomyopathy associated with electrical conduction disturbances caused by abnormal Cx43 remodelling (Smyth et al., 2010; Macquart et al., 2018). Many proteins that regulate cardiomyocyte contraction have short half-lives, making efficient production and accurate localisation of these regulatory proteins crucial. For example, Cx43 has a half-life between 2 and 5 h, indicating that the entire gap junction renews multiple times daily (Epifantseva and Shaw, 2018). Other ion channels, such as a voltage-gated Na^+^ channel Na_v_1.5 and an inwardly rectifying K^+^ channel Kir2.1, exhibit a similar turnover rate (Ponce-Balbuena et al., 2018). With both K^+^ and Na^+^ being direct regulators of cardiac action potential, efficient assembly of these ion channels through microtubule-mediated molecular shuttling is essential for maintaining cardiac homeostasis.

Microtubules also regulate Ca^2+^-handling proteins, affecting excitation–contraction coupling. Microtubule-dependent alignment of mitochondria and the sarcoplasmic reticulum ensures proper Ca^2+^ release in cardiomyocytes (Miragoli et al., 2016; Uchida et al., 2022). Displacement of these organelles can cause aberrant Ca^2+^ release, interfering with synchronous cardiac contraction. In Duchenne muscular dystrophy, microtubule network disruption increases reactive oxygen species production, which alters Ca^2+^ signalling and excitation–contraction coupling (Khairallah et al., 2012). Similarly, microtubules are crucial mediators of PIEZO2 mechanotransduction (Chang and Gu, 2020); misregulation of PIEZO channels in cardiomyocytes is linked to disease phenotypes, such as cardiac hypertrophy and pulmonary hypertension (Coste and Delmas, 2024).

Microtubule-mediated protein misregulation can cause disruptions in t-tubule formation, further contributing to Ca^2+^ mishandling and the development of associated cardiomyopathies (Prins et al., 2016; Kwan et al., 2023). Additionally, β-adrenergic receptors (β-ARs), which regulate cAMP signalling and modulate cardiac function and metabolism, rely on the microtubule network for proper localisation. In the failing heart, both β1-AR and β2-AR are redistributed; β2-AR redistribution is particularly dependent on microtubules and t-tubule integrity (Kwan et al., 2023). In summary, the role of microtubules in intracellular transport and signalling modulation is essential for healthy cardiomyocyte function, and alterations in this system can lead to cardiac disease pathologies.

## Microtubule network, mechanobiology and cardiac disease

In response to mechanical stressors associated with cardiovascular diseases (such as myocardial infarction, cardiac amyloidosis, cardiac hypertrophy and dilated cardiomyopathy), adult cardiomyocytes undergo extensive structural, electrical and functional remodelling. Key mechanical drivers of this pathological adaptation include pressure or volume overload (Kishimoto et al., 2023), fibrosis and ECM hardening (Majkut et al., 2013), arrhythmias and junction remodelling (Le Dour et al., 2022).

Following a myocardial infarction, the mechanobiological properties of the myocardium change through multiple mechanisms, ultimately reducing contractile dynamics and increasing the risk of arrhythmias. These changes vary across the progression of the disease. In the acute phase, the microtubule cytoskeleton within individual cardiomyocytes undergoes rapid modifications driven by as-yet unidentified upstream factors, which enhance passive microtubule-generated forces and alter contractile function (Yu et al., 2021). Because the adult heart has limited regenerative capacity, remodelling in response to injury leads to further heart complications later in life, even after surviving the acute myocardial infarction. At the tissue level, fibrotic remodelling increases myocardial stiffness and forms a dense, non-conductive and non-contractile scar. This scar tissue prevents local contraction and inhibits the propagation of electrical signals (Engler et al., 2008; Olvera et al., 2020). At the cellular scale, this ECM remodelling disrupts cytoskeletal tension, alters tissue stiffness and restricts cardiomyocyte movement, leading to aberrant signalling that can drive pathological progression (Jaalouk and Lammerding, 2009).

Beyond ischemic injury, other mechanical stressors, including chronic pressure overload and ageing, trigger distinct remodelling pathways. Changes in tissue mechanical properties alter the forces and pressures received by embedded cardiomyocytes. Pressure overload reduces contractility and triggers upregulation of mechanosensitive genes including *NPPB*, resulting in contractile dysfunction (Kishimoto et al., 2023). For this reason, B-type natriuretic peptide, a hormone that is secreted by heart ventricles in response to various forms of cardiac pathology including abnormal stretch, is widely used as a clinical biomarker of heart dysfunction. Pressure overload-induced cardiac hypertrophy is regulated in part by the mechanosensor PIEZO1 (Yu et al., 2022), a stretch-activated ion channel that initiates the hypertrophic response to pressure overload by disrupting intracellular Ca^2+^ homeostasis (Zhang et al., 2021). During ageing, progressive myocardial stiffening requires greater force generation by contractile cells (Lieber et al., 2004; Hersch et al., 2013).

β-ARs, important transmembrane proteins in cardiomyocyte mechanotransduction, trigger intracellular signalling cascades that can modulate heart rate and blood pressure. Following myocardial infarction, these receptors become mislocalised in cardiomyocytes (Kwan et al., 2023). Loss of t-tubules, where β2-ARs normally reside, results in a redistribution of β2-ARs to other cell surfaces (Nikolaev et al., 2010; Kwan et al., 2023). This redistribution results in aberrant cyclic adenosine monophosphate (cAMP) signalling, which can impact heart rate and contraction.

Abnormal remodelling of intermediate filaments can further disrupt intracellular organisation, contributing to the development of dilated cardiomyopathies. For example, mutations in the lamin A/C-encoding gene (*LMNA*) decrease α-tubulin acetylation, causing left ventricle dilation and atypical Cx43 localisation (Le Dour et al., 2022). Lamins are crucial to nuclear and organelle tethering, with alterations in LMNA interfering with mechanosensitive gene expression leading to human diseases such as Emery–Dreifuss muscular dystrophy and dilated cardiomyopathies (Lammerding et al., 2004). Other intermediate filaments, such as desmin, are often localised at Z-discs in cardiomyocytes; loss of proper Z-disc anchoring results in inhibited stretch sensing and can lead to dilated cardiomyopathies (Knöll et al., 2002).

In summary, the disruption of essential mechanotransducers compromises mechanical integrity, cell organisation, intracellular cell signalling and extracellular ion exchange, directly contributing to cardiomyopathy.

## Conclusions

As discussed in this Review, mechanobiology is an innate feature of cell biology that shapes how cells explore, sense and respond to their physical environment. This is particularly important for cardiomyocytes, which are embedded in the myocardium – one of the most mechanically active tissues in the body. Consequently, cardiomyocytes must handle rapidly changing forces, frequencies and geometries.

Mechanobiology is important for cardiac function from the earliest stages of development, with mechanotransduction pathways tightly regulating cardiomyocyte development, maturation and organisation. In adults, mechanobiology is essential for maintaining healthy cardiac function, including mechanosensitive regulation of important ion channels. Disruption of these pathways can alter cardiomyocyte contraction and lead to dangerous disease phenotypes at the organ level. Microtubules in cardiomyocytes are increasingly recognised as essential players for cardiac mechanobiology. Future research will need to define more precisely how microtubule networks serve as dynamic mechanosensors, integrating mechanical load with biochemical signalling to drive adaption or disease. A key future direction will be the development of therapies that selectively modulate microtubule stability or their interactions with mechanotransduction hubs, to correct maladaptive remodelling in heart disease.

Cardiomyocytes do not live in isolation. The myocardium is composed of many cell types that contribute to cardiomyocyte function and overall cardiac output (Box 1). Exciting engineering approaches are being investigated to leverage mechanotransduction pathways to better understand cardiomyocyte behaviour and disease responses (Box 2). The understanding of cardiomyocyte mechanobiology can be used to develop novel *in vivo* therapeutic strategies and improve the physiological relevance and maturity of *in vitro* cardiac models.

Box 1. Mechanobiology of other cardiovascular cells Cardiomyocytes do not operate in isolation; they are part of a diverse multicellular tissue that contains cardiac fibroblasts (Pesce et al., 2023), epicardial adipocytes (Liu et al., 2022), smooth muscle cells (Johnson et al., 2024), endothelial cells, mesenchymal stem cells (Mayourian et al., 2018; Raman et al., 2022) and resident macrophages (Suku et al., 2022). Although cardiomyocytes account for only 20–30% of cardiac cell numbers, their large size means they comprise 70–85% of myocardial volume (Guo and Pu, 2020). The remaining 70–80% of cardiac cells perform different functions that can significantly impact cardiomyocyte mechanobiology.

Fibroblasts are rapid producers of ECM, which provides tissue integrity and alters tissue stiffness. After myocardial infarction, ischemic tissue is replaced by fibrotic scar that has increased stiffness, reduced contractility and limited electrical conduction (Olvera et al., 2020). These biomechanical changes contribute to long-term heart failure in many survivors. Similarly, adverse inflammatory remodelling in epicardial adipocytes can alter myocardium mechanics in cardiovascular disease (Liu et al., 2022). Macrophages, in addition to their immunomodulatory roles, have emerged as enablers of electrical conduction that maintain excitation–contraction coupling (Suku et al., 2022). Supplementing engineered cardiac tissues with macrophages improves tissue maturity, enhances long-term vascularization and improves contractile performance (Landau et al., 2024; Lock et al., 2024; Suku et al., 2024 preprint). Notably, engineered tissues composed solely of cardiomyocytes do not achieve maximal mechanical performance, highlighting the essential contributions of nonmyocyte cell populations to cardiomyocyte function (Munawar and Turnbull, 2021).

The specific mechanisms underlying nonmyocyte contributions to cardiomyocyte function remain unexplored (Munawar and Turnbull, 2021). However, these effects can be partly explained by alterations in the mechanical environment of the myocardium and the activation of mechanotransduction pathways in cardiomyocytes.

Box 2. Engineering cardiomyocyte biology to improve mechanical function Bioengineering and biomanufacturing are powerful tools for fabricating complex *in vitro* models, generating biomimetic structures and producing novel therapeutic technologies. These techniques, including tissue engineering, 3D bioprinting, organoids and biomimetic scaffold engineering, are rapidly emerging and evolving (Gao et al., 2024; Murphy et al., 2024; He et al., 2025) and can be focused at the cellular, tissue or whole-organ level.

At the cellular level, engineering approaches have been applied to mature cardiomyocytes. Physically, cardiomyocyte shape and orientation have been modulated through substrate engineering, including 2D surface coating (Batalov et al., 2021) and 3D microgroove patterning (Al Sayed et al., 2024). These approaches constrain adhesion and spreading, guiding cells towards an elongated, more mature and rod-shaped phenotype. Engineering their biochemical microenvironment is equally important. Hydrogels are used in 3D culture to match ECM-like mechanical properties (Nunes et al., 2013; Turnbull et al., 2018), electrical conditioning recapitulates the electromechanical signalling experienced *in vivo* (Radisic et al., 2004; Godier-Furnémont et al., 2015), and maturation medium is formulated to mirror physiological metabolite concentrations and regulate metabolic fate (Feyen et al., 2020).

At the tissue and organ levels, myocardial infarction disrupts contractile function because scar tissue dampens both electrical conduction and physical contraction (Engler et al., 2008). To promote regeneration, strategies include direct cell injection and scaffold-based cell delivery. Wet-adhesive hydrogel patches, for example, have been used to deliver stem cells and small molecules to the infarcted myocardium (Wu et al., 2023), providing therapeutic delivery alongside mechanical and potentially electrical support (Mei and Cheng, 2020).

Cell-free approaches have also emerged to regulate myocardial mechanical function. Conductive mechanical patches engineered to match the auxetic (expands in direction perpendicular to the load) and synclastic (all sides curved toward the same direction) properties of the human heart can restore electrical and physical properties (Kapnisi et al., 2018; Olvera et al., 2020) and can also enable real-time monitoring (Yu et al., 2023). Modulating immune response through biopolymers such as itaconate, a macrophage-derived metabolite, can improve cardiac function and disease response (Davenport Huyer et al., 2021). Finally, delivering RNAs (modified mRNA or miRNA) that have been identified as drivers of paracrine-mediated cardiac tissue repair, offers another promising for cell-free therapeutic avenue (Mayourian et al., 2018; Laggerbauer and Engelhardt, 2022).
